# Posturographic Standards for Optimal Control of Human Standing Posture

**DOI:** 10.5114/jhk/159452

**Published:** 2023-01-20

**Authors:** Janusz W. Błaszczyk, Monika Beck

**Affiliations:** 1 Department of Human Motor Behavior, The Jerzy Kukuczka Academy of Physical Education, Katowice, Poland.

**Keywords:** static posturography, postural control, stability, postural sway

## Abstract

Static posturography is a simple non-invasive technique commonly used in contemporary labs and clinics to quantify the central nervous system adaptive mechanisms involved in the control of posture and balance. Its diagnostic value, however, is quite limited due to the lack of posturographic standards for the stable posture. To solve this problem, in this research, we aimed to establish reference values for the stable human posture using our novel parameters of static posturography including the sway anteroposterior directional index (DIAP), the mediolateral directional index (DIML), the stability vector amplitude (SVamp), and the stability vector azimuth (SVaz). Towards this end, in a population of young (mean age 22 yrs), healthy able-bodied volunteers (50 males and 50 females), trajectories of postural sway, based upon the center-of-pressure (COP), were assessed. The experiment consisted of ten 60 s trials that were carried out 5 times while subjects were standing quietly on the force plate with eyes open (EO test) and 5 times with eyes closed (EC test). Results showed that in young healthy subjects, regardless of gender, the basic variables of COP remained at the following levels: SVamp = 9.2 ± 1.6 mm/s, SVaz = 0.9 ± 0.1 rad, and directional indices DIAP = 0.7 ± 0.05, DIML = 0.56 ± 0.06. Some of the measures were sensitive to visual input (EC trials), and showed a weak to moderate correlation with anthropometric features. These measures can be recommended as reference values that characterize the most stable erect posture.

## Introduction

The control of human standing posture can be modeled as a process of positioning the body's center of gravity (COG) within the center of the base of support (BOS). Due to several limitations of neuromuscular control, the output of control is not a single point, but rather randomly oscillates within a limited area. The quality of postural control can be assessed using characteristics of the system output, e.g., trajectories of the COG during a quiet stance. In force-plate posturography, trajectories of the center of pressure (COP) that contain information on COG displacements are used to assess postural stability (Błaszczyk, 2008). To solve these problems, several dedicated standardized measures of the COP have been developed in our laboratory (Błaszczyk, 2008, 2016; Błaszczyk et al., 2014).

Usually, it is a rather demanding task to find straightforward relationships between the COP spontaneous oscillations and the stability of human standing posture (Mizrahi et al., 2006; Pasma et al., 2017; Rougier, 2008; Yamamoto et al., 2015). The COP signal is composed of the COG sway trajectory (frequency band 0–0.5 Hz) superimposed on stabilizing force moments (frequency band 0.5–5 Hz in young healthy subjects) (Błaszczyk, 2008). This complex signal can be easily accessed using a force-plate posturography, however, in this method, the COP is usually contaminated with sampling noise, which needs to be removed by low-pass filtering e.g., at 10 Hz (Błaszczyk, 2008; Błaszczyk et al., 2016).

In static posturography, the process of erect posture stability control is modeled as maintaining the COP within the BOS at a reference position, which is considered the set point of postural control (Błaszczyk et al., 1994; Błaszczyk, 2016). In the maximally stable control, the COP set point should be located in the middle of the BOS (Błaszczyk et al., 1994). Such a set point provides an equal probability to recover balance in case of unpredicted perturbation. In a quiet stance, the BOS envelope approximates the area of stability, thus a minimum distance between the COP set point and stability borders can approximate the stability radius (Bingham and Ting, 2013). Consequently, the stability vector amplitude (SVamp), which is equal to the mean COG sway velocity, determines the maximal time limit when balance recovery is possible (Błaszczyk, 2016). Unfortunately, due to anatomical and physiological asymmetries as well as lateralization of the human body, the direction of the COP displacement changes in a random fashion and to assess reliably postural stability, the SVaz should be always taken into account (Błaszczyk, 2016; Błaszczyk et al., 2020). Importantly, changes in the mediolateral (ML) plane are often more pronounced in deficient postural control (Błaszczyk et al., 2016; Maki et al., 1996). This observation allows us to recommend the directional indices (DIAP, DIML) and directional sway ratios as the main veridical measures of postural stability (Błaszczyk, 2008; Błaszczyk et al., 2014).

The main drawback of static posturography is the dependence of measured sway variables on the measurement methodology, e.g., the duration of a trial and signal sampling frequency. For this reason, we have developed several COP parameterization methods based on normalized measures which are independent of the measurement conditions. Particularly, directional COP indices (DIAP and DIML) (Błaszczyk et al., 2014) occurred to be reliable sway measures that allow for improving postural stability assessment (Błaszczyk et al., 2016). To establish standards of maximally stable postural control, in this research, we focused on the set of 4 normalized COP variables (DIAP, DIML, and SVamp, SVaz) which were collected in a large group of young, able-bodied adults, whose sensory and neuromuscular development had already been completed (Assaiante et al., 2014).

## Methods

The study protocol was approved by the Senate Ethics Committee (1/2011). The experiments were conducted following the principles of the Declaration of Helsinki and all participants provided their written informed consent before the commencement of the study. The experimental group consisted of 100 young able-bodied subjects (50 female and 50 male). Their age ranged from 19 to 28 years (mean age 21 ± 2 years). To assess the optimal characteristics of postural control, center-of-pressure (COP) trajectories while standing quiet were recorded and analyzed. During the experimental session, each participant completed five 60-s trials with ‘eyes open’ (EO) and then five with ‘eyes closed’ (EC). The measurements were made on the force plate (Type 9281C Kistler Group, Switzerland). The COP trajectories were sampled at 40 Hz and stored for offline analysis. In particular trials, participants could modify their position on the force plate, thus each position was characterized by a different mean COP value which caused an offset in the data. Therefore, before filtering the offset was removed. Signals were then filtered with a low-pass filter with a 5 Hz cut-off frequency (Cheby II, Matlab MathWorks USA).

Based on filtered trajectories, COP sway velocities in main anatomical planes, i.e., anteroposterior (*VAP*) and mediolateral (*VML*), as well as total velocity (*VTOT*), were computed. The Sway Vector amplitude (*SVamp*) and Sway Vector azimuth (*SVaz*) were computed according to the following formula (Błaszczyk, 2016):


$$SVamp=V_{TOT}\text{ and SVaz = arctan }\frac{V_{AP}}{V_{ML}}\text{ (1)}$$

Anteroposterior (*DIAP*) and mediolateral (*DIML*) directional sway indices were defined as the ratio of anteroposterior or mediolateral COP velocities and the total COP velocity (Błaszczyk et al., 2014):


$$DIAP=\frac{V_{AP}}{V_{TOT}}\text{ (2)}$$

$$DIML=\frac{V_{ML}}{V_{TOT}}\text{ (3)}$$

Sway variables for each group and experimental conditions were presented by means and standard deviations. Statistical significance for the data collected during standing was analyzed for the main effects of vision and trial using a twoway repeated-measures ANOVA (Statistica v.10, StatSoft U.S.A.). Relationships between sway characteristics and directional sway measures were evaluated by Pearson's (r) correlation. Statistical significance was accepted at *p* ≤ 0.05.

## Results

### Sway Velocity (SVamp) and Sway Vector Azimuth (SVaz)

**Figure 1:**
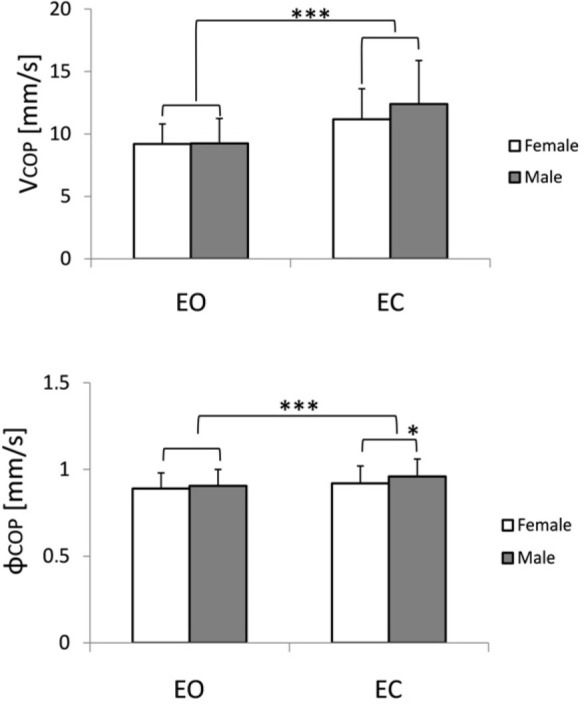
Sway Vector variables: SVamp = VCOP (upper panel), and azimuth SVaz = φCOP as measured in young healthy adults (n = 100) while standing quietly with eyes open (EO) and eyes closed (EC). * p ≤ 0.01, *** p ≤ 0.0001

ANOVA revealed a significant effect of vision (F_1,98_ = 326.7, *p* ≤ 0.000001) for COP velocity that represents in our model SV amplitude (SVamp). Additionally, gender x vision interaction was significant (F_1,98_ = 16.63, *p* ≤ 0.005). Generally, the mean COP velocity in both groups remained almost at the same level (9.19 ± 1.6 mm/s and 9.24 ± 2 mm/s in the female and male groups, respectively). Eyes closure resulted in an increase in the COP velocity up to 11.2 ± 2.45 mm/s in females, and to 12.38 ± 3.48 mm/s in males. The results of this analysis are summarized in Figure 1A.

Sway Vector azimuth (SVaz) showed no gender difference (*p* ≤ 0.13), whereas the effect of vision (F_1,98_ = 82.1, *p* ≤ 0.0001) and gender-vision interaction (F_1,98_ = 7.97, *p* ≤ 0.01) differed significantly. Under full sensory control (EO trials), the sway vector azimuth remained at the level of 0.89 ± 0.09 rad, and 0.91 ± 0.09 rad in females and males, respectively. Eyes closure (EC) resulted in an increase in SVaz to 0.92 ± 0.1 rad in the female group and up to 0.96 ± 0.1 rad in the male group (Figure 1B).

### The Velocity of Anteroposterior (VAP) and Mediolateral (VML) COP Sway

**Figure 2:**
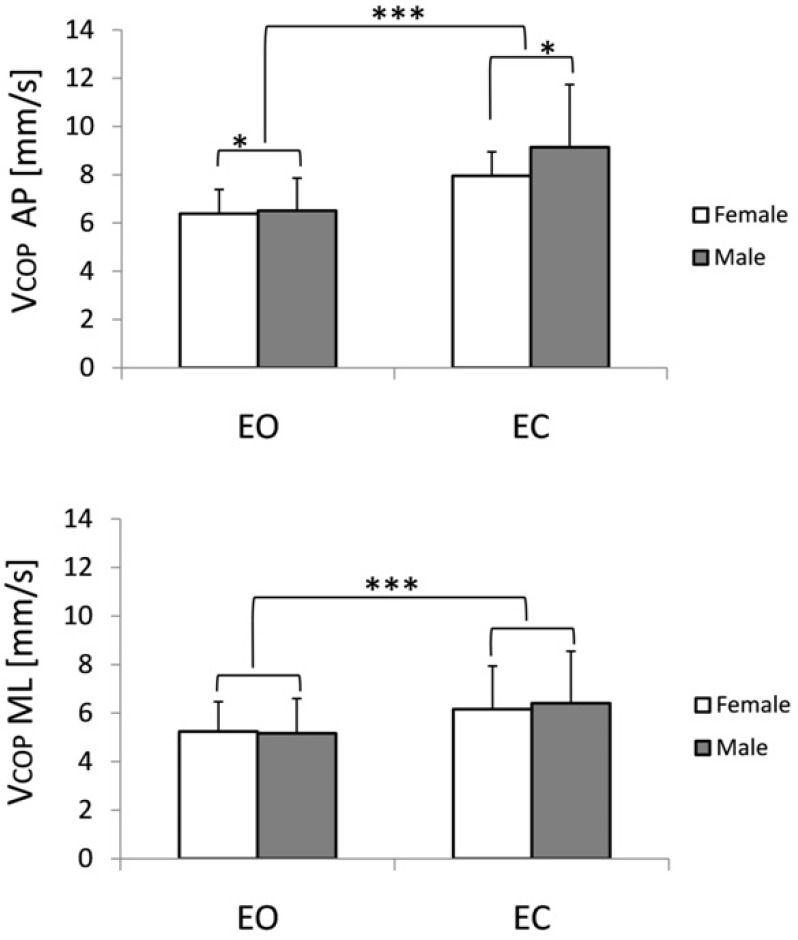
Impact of visual input on anteroposterior VCOP AP (upper panel) and mediolateral (VCOP ML) sway velocity in the male and female groups of young able-bodied subjects while standing with eyes open (EO) and eyes closed (EC). * p ≤ 0.01, *** p ≤ 0.0001

Analysis of variance with gender and vision as independent factors documented the significant impact of both factors on the mean VAP velocity while tested with (EO) and without (EC) visual input. Generally, performing the test with eyes closed resulted in an increased VAP component, which in the female group was 6.39 ± 1.0 mm/s (EO) and 7.95 ± 1.6 mm/s (EC), whereas in the male group tested with eyes open, the VAP velocity remained at the level of 6.5 ± 1.35 mm/s and increased up to 9.14 ± 2.6 mm/s in EC trials. Both effects were statistically significant: F_1,98_ = 4.1, *p* ≤ 0.05 (males vs. female), and the impact of vision on VAP velocity was more pronounced (F_1,98_ = 267.8, *p* ≤ 0.00001). Group x vision interaction was also significant (F_1,98_ = 17.5, *p* ≤ 0.0001). The results are depicted in Figure 2A.

Less significant changes were observed in the mediolateral COP velocity. Analysis of variance showed the main effect of vision only (F_1,98_ = 143.8, *p* ≤ 0.00001) for the VML. Thus, the increase in the VML observed in response to eye closure was very similar in both groups: 5.2 ± 1.2 mm/s and 5.2 ± 1.4 in the EO test, females and males, respectively. Also, an increase in ML sway, while tested without vision (EC test), remained at a similar level i.e., 6.2 ± 1.8 mm/s in the female group, and 6.4 ± 2.1 mm/s in the male group. The results are presented in Figure 2B.

### Anteroposterior (DIAP) and Mediolateral (DIML) Directional COP Indices

**Figure 3:**
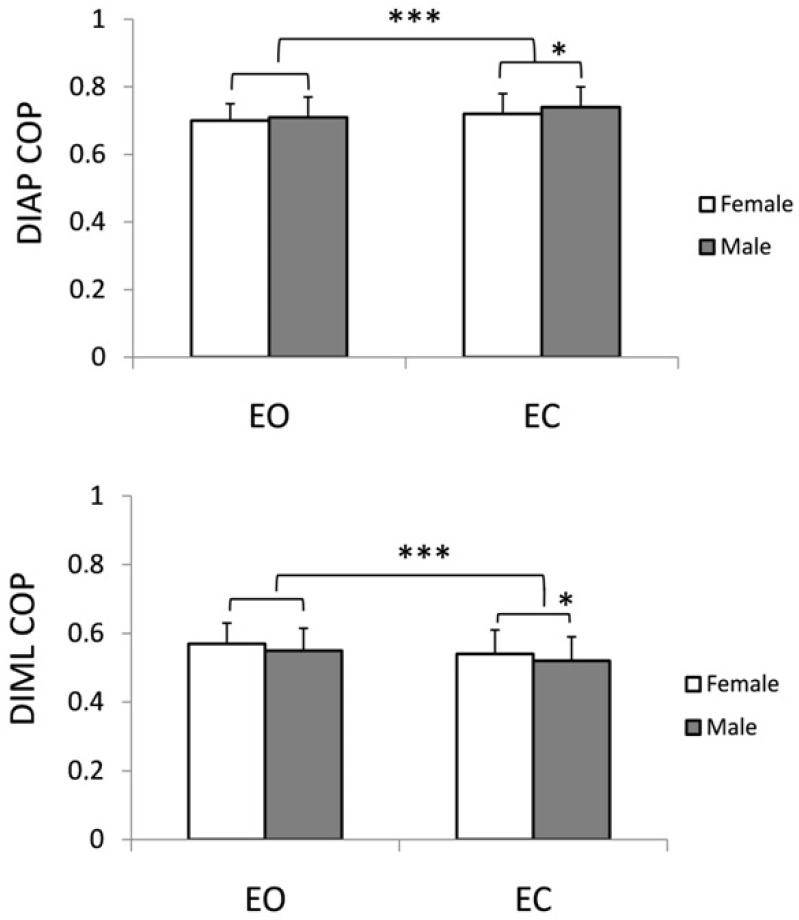
Changes in directional sway indices (anteroposterior DIAP, and mediolateral DIML) in young subjects standing with eyes open (EO) and eyes closed (EC). * p ≤ 0.01, *** p ≤ 0.0001

In the female group, while standing quiet, the eyes closure resulted in an increase in the DIAP from 0.70 ± 0.05 in the EO trial to 0.72 ± 0.06 in the EC condition (for details see Figure 3A). A similar effect of vision on DIAP was observed in the male group. Here DIAP value increased from 0.71 ± 0.06 in full input conditions up to 0.74 ± 0.06 when the visual input was excluded from the control of posture (F_1,98_ = 77, *p* ≤ 0.00001). Interaction of both factors (gender and vision) also reached the level of significance (F_1,98_ = 6.17, *p* ≤ 0.02).

The impact of excluded visual input on COP sway was also documented by a decrease in DIML value from 0.57 ± 0.06 rad, and 0.55 ± 0.06 in the EO trial in female and male groups, respectively, to 0.54 ± 0.07 (female group), 0.56 ± 0.06 to 0.52 ± 0.07 in males while tested with EC (Figure 3B). Also, gender-by-vision interaction was statistically significant (F_1,98_ = 8.3, *p* ≤ 0.005). The main results are gathered in Table 1.

**Table 1:** Reference values of the center-of-pressure (COP) characteristics (mean ± s.d.) in male and female participants for a 60-s habitual stance with ‘eyes open’(EO) and ‘eyes closed’ (EC).

Group Reference values	Female		Male	
	EO	EC	EO	EC
SVamp(mm/s)	9.2 ± 1.6	11.2 ± 2.4	9.2 ± 2.0	12.4 ± 3.5
SVaz(rad)	0.9 ± 0.1	0.9 ± 0.1	0.9 ± 0.1	1.0 ± 0.1
DIAP	0.7 ± 0.1	0.7 ± 0.1	0.7 ± 0.1	0.7 ± 0.1
DIML	0.6 ± 0.1	0.5 ± 0.1	0.6 ± 0.1	0.5 ± 0.1

### Correlations Analysis

Particularly noteworthy is the fact that variables characterizing body sway while standing quiet with eyes open and with eyes closed were correlated (*p* ≤ 0.05) with participants’ body anthropometric features such as body height, BMI, and the BOS size. The results of this analysis are summarized in Table 2. In particular, the AP and ML directional indices and SVaz both in EO and EC, showed a moderate correlation with most of the anthropometric features. Some sway measures recorded for EO and EC conditions in young healthy subjects also showed a moderate to strong correlation (Table 3).

**Table 2:** Results of correlation analysis (Pearson R test) between selected body anthropometric measures including the base of support (BOS), in 50 young female (F) and 50 male (M) subjects and major COP measures while standing quiet with eyes open (EO) and eyes closed (EC).

r *p* ≤0.05		EO					EC				
		SVamp	SVa		DIAP	DIML	SVamp	SVaz		DIAP	DIML
Body Height	F	0.26	−0.43		−0.43	0.43	0.35	−0.40		−0.40	0.38
	M	0.20	−0.40		−0.40	0.39	0.31	−0.25		−0.25	0.25
BOS Width	F	−0.26	0.45		0.44	−0.46	−0.30	0.51		0.50	−0.51
	M	NS	0.26		0.25	−0.26	NS	0.41		0.41	−0.40
BOS Length	F	NS	NS		NS	NS	NS	NS		NS	NS
	M	NS	−0.34		−0.34	0.34	NS	−0.20		−0.20	0.21
BMI	F	−0.38	0.41		0.40	−0.42	−0.29	0.46		0.45	−0.47
	M	NS	0.31		0.32	−0.31	NS	0.23		0.23	−0.24

**Table 3:** Results of correlation analysis (Pearson r test) between COP measures while standing quiet with eyes open (EO) and eyes closed (EC) in n = 100 young subjects.

r *p* ≤0.05	EO				EC					
	VAP	VML	DIAP	DIML	SVamp	SVaz	VAP	VML	DIAP	DIML
SVamEO	0.91	0.91	−0.37	0.37	0.89	−0.27	0.81	0.84	−0.27	0.27
SVazEO	NS	0.71	1	−1	−0.34	0.87	NS	−0.66	0.88	−0.88
DIAPEO	NS	−0.71	1	−1	−0.34	0.87	NS	−0.66	0.87	−0.87
DIMLEO	NS	0.71	−1	1	0.34	−0.88	NS	0.66	−0.88	0.88

Changes in directional sway indices (anteroposterior DIAP, and mediolateral DIML) in young subjects standing with eyes open (EO) and eyes closed (EC). * p ≤ 0.01, *** p ≤ 0.0001

## Discussion

Postural control is an adaptive, multi-input, dynamic system of which robustness and effectiveness depend on many physiological and anatomical factors (Chadges et al., 2013; Feldman and Levin, 2016; Muehlbauer et al., 2015; Schmuckler and Tang, 2019). The stability of human erect posture is controlled in the sagittal plane by ankle joint stabilizers, while in the frontal plane, control mainly relies on a hip (load/unload) mechanism (Winter et al., 1996). The main task of fully efficient postural control is to maintain the COG roughly in the center of the base of support. Such functional symmetry allows the recovery of equilibrium with the same probability regardless of the direction of the disturbance. Regarding postural sway as a random perturbation, its magnitude and directional characteristics need be taken into account for optimal postural control. In this context, both directional sway measures, i.e., DIAP and DIML, as well as variables of the stability vector, point to the weakest points (direction and stability radius) of postural stability control. Here in the search for the most optimal control, we analyzed body sway in young adults known for the most stable posture.

The stability of human erect posture is determined by several factors, among which the main is the size of the stability area. The area is limited by irregular and asymmetric borders. Generally, the stability area can be described by AP and ML stability radii (Bingham and Ting, 2013; Hindrichsen and Pritchard, 1986). As could be expected from the structure of the human body inverted pendulum in young able-bodied subjects to maintain the most stable posture, the control system allocates more effort to control AP stability. In the frontal plane, most COP oscillations are observed (Borg et al., 2007; Maurer and Peterka, 2005). However, the probability of instability, which means the probability of uncontrolled crossing of rear, frontal or lateral borders of stability during a quiet stance, is very low. Even excluding the visual input (EC trials) does not result in a significant increase in the COP oscillations, thus in the substantial changes in postural stability.

This study also supports the notion that the stability vector (SV) can be a useful measure of postural stability (Błaszczyk, 2016). The SV is an extension of the applied control theory concepts of the stability radius. Its magnitude is equal to the mean COP velocity. To date, the mean COP velocity has been assumed as a gold standard for postural stability control (for review see Raymakers et al., 2005). The low magnitude of the stability vector, as observed in our participants, reflects decreased uncertainty in postural control and maximizes the time for balance recovery action in case of perturbation. In this context, the human standing posture is rather stable and robust. The relatively low AP velocity of COP displacements (6.5 mm/s EO and 8.5 mm/s EC) does not pose a threat to stability. The uncontrolled drift of the COP with this speed toward the anterior stability border would last at least a couple of seconds, giving enough time to detect instability and successfully recover equilibrium (Błaszczyk et al., 1993, 1994).

The stable erect posture may be characterized by the natural proportion between the AP and ML sway. The proportion can be assessed based upon the DIAP and DIML, as well as the SV azimuth. Only increased ML oscillations (which correspond in our research to an increased DIML or a decreased SVaz) may indicate a decline in postural stability (Błaszczyk, 2016). In young healthy subjects standing with their eyes open, directional indices of values are very stable (DIAP=0.7 and DIML = 0.56). Excluding visual input, however, which impoverished control, resulted in slight, but statistically significant changes in the DIML only (dropped to 0.5), while no changes in DIAP were observed. The ratio of both directional indices determines the azimuth of the stability vector, which points to the most probable direction of the postural disturbance. The SVaz = 0.9 rad (51.6 deg) corresponds to the optimal level of interaction between the AP and ML control. In young subjects, the SVaz is almost independent of visual input (0.9 rad EO and 0.95 rad EC). However, trials with eyes closed reveal some subtle differences between male and female groups in the control of upright posture. In the male group, the SVaz EC increased to 1 rad, which can be explained by different body anthropometry of males, especially their greater body weight and height. As documented by our results of correlation analysis, postural stability, in addition to the size of the support surface, depends also on body height and body mass, which are the main determinants of the inverted pendulum model.

In summary, postural control in young healthy adults is robust and effective. The most stable human erect posture is characterized by limited sway velocity (SVam), almost constant values of directional indices (DIAP, DIML), and the sway vector azimuth (SVaz). Results of the current study allow to recommend these measures as a standard for optimal and robust postural control of human standing posture.
